# Age-related changes in K_v_4/Shal and K_v_1/Shaker expression in *Drosophila* and a role for reactive oxygen species

**DOI:** 10.1371/journal.pone.0261087

**Published:** 2021-12-21

**Authors:** Maximiliano J. Vallejos, Abdunaser Eadaim, Eu-Teum Hahm, Susan Tsunoda

**Affiliations:** Department of Biomedical Sciences, Colorado State University, Fort Collins, Colorado, United States of America; University of Modena and Reggio Emilia, ITALY

## Abstract

Age-related changes in ion channel expression are likely to affect neuronal signaling. Here, we examine how age affects K_v_4/Shal and K_v_1/Shaker K^+^ channel protein levels in *Drosophila*. We show that K_v_4/Shal protein levels decline sharply from 3 days to 10 days, then more gradually from 10 to 40 days after eclosion. In contrast, K_v_1/Shaker protein exhibits a transient increase at 10 days that then stabilizes and eventually declines at 40 days. We present data that begin to show a relationship between reactive oxygen species (ROS), K_v_4/Shal, and locomotor performance. We show that K_v_4/Shal levels are negatively affected by ROS, and that over-expression of Catalase or RNAi knock-down of the ROS-generating enzyme, Nicotinamide Adenine Dinucleotide Phosphate (NADPH) Oxidase (NOX), can attenuate the loss of K_v_4/Shal protein. Finally, we compare levels of K_v_4.2 and K_v_4.3 in the hippocampus, olfactory bulb, cerebellum, and motor cortex of mice aged 6 weeks and 1 year. While there was no global decline in K_v_4.2/4.3 that parallels what we report in *Drosophila*, we did find that K_v_4.2/4.3 are differentially affected in various brain regions; this survey of changes may help inform mammalian studies that examine neuronal function with age.

## Introduction

Aging has long been associated with a decline in cognitive and motor function, and this is a phenomenon that is conserved across species [1–6]. Changes in ion channel expression and function that occur with age are likely to compromise signaling and may be a contributing factor to the progressive loss of motor and cognitive function. Multiple ion channels and neurotransmitter receptors, which shape signaling in the nervous system, have been shown to be affected by age. For example, levels of mRNA encoding subunits of AMPA and NMDA receptors have been shown to be reduced by half in the prefrontal cortex of older adults (> 40 years) compared to younger adults [7]. In rodents, AMPA receptor subunits, including GluR1, have also been shown to be lost with age in some brain regions [8–10]. Mouse NMDA receptor subunits NR1 and NR2B mRNA and protein were shown to be reduced in the aged hippocampus [11–14]. Interestingly, with age, greater levels of stimulation are required for inducing long-term potentiation (LTP) (reviewed in [15]), and spatial memory and motor function have been correlated with levels of NMDA receptors [16, 17]. GABA receptors have also been shown to be affected by age, with altered levels of subunit mRNA and protein levels in some brain regions [18, 19].

Voltage-dependent K^+^ channels have been reported to be affected by age as well. K_v_1.1 and K_v_1.2 channel protein levels are enhanced with age in both cerebellar output neurons and cochlear nuclei of rats [20, 21], and K_v_3.1 protein levels have been reported to decline with age in the posterior ventral cochlear nucleus in the rat auditory system [20]. One report has shown age-related hyperexcitability in CA3 pyramidal neurons that is due to an enhancement in the A-type K^+^ current, and a concurrent increase in K_v_4.2 and K_v_4.3 [22].

With age, the accumulation of reactive oxygen species (ROS) has been proposed as a key factor contributing to how some ion channels are affected by age. ROS have been shown to affect gene expression and proteostasis at multiple levels, from synthesis to degradation. For example, studies have suggested that increased levels of ROS cause damage to nucleic acids, and even preferentially to promotor regions of genes [7], leading to a decline in some mRNAs with age [23]. ROS also lead to protein oxidation, resulting in protein dysfunction and often aggregation [24, 25]. In processes conserved across species, the K^+^ channel, K_v_2.1, has been shown to undergo protein oxidation by ROS, inducing oligomerization of K_v_2.1 channels [26, 27] that renders them non-functional, promoting hyperexcitability, and impairing working memory [27–29].

In this study, we show that in *Drosophila*, K_v_4/Shal protein levels decline sharply in the early life of the fly with a concurrent increase in K_v_1/Shaker protein. K_v_4/Shal protein levels then exhibit a continual decline to levels less than 20% by 40 days after eclosion (AE). This substantial loss in K_v_4/Shal protein is interesting since K_v_4/Shal channels are the most highly conserved subfamily of voltage-dependent K^+^ channels with 84% amino acid identity between flies and mice [30], and K_v_4/Shal channels across species have been shown to modulate Hebbian and homeostatic forms of synaptic plasticity, and contribute to functions such as locomotion and cognition. All K_v_4/Shal channels, from invertebrates to mammals, have been shown to encode fast transient A-type K^+^ currents [30–33]. In neurons, K_v_4/Shal channels are localized to somato-dendritic sites, where they play important roles in modulating neural activity, regulating the integration of high-frequency trains of synaptic input [34], modulating incoming miniature excitatory post-synaptic currents (mEPSCs) [35], regulating backpropagating action potentials (bAPs) [36–38], and contributing to long-term potentiation (LTP) [35, 38, 39]. In *Drosophila*, K_v_4/Shal channels have been shown to regulate the onset and frequency of AP firing [40], modulate mEPSCs and synaptic homeostasis [41, 42], and critically contribute to locomotor and learning/memory performance in both physiological and pathological conditions [40, 43]. We also examine how age affects locomotor performance, and how over-expression of *K*_*v*_*4/Shal* or *K*_*v*_*1/Shaker* affects this performance. We present evidence that the age-dependent accumulation of ROS may contribute to the decline of K_v_4/Shal protein.

## Materials and methods

### Fly stocks

*w*^*1118*^ or genetic background strains were used as control lines in this study. Fly strains used include: Canton-S (Bloomington *Drosophila* Stock Center, Stock 64349), *w*^*1118*^, *SK*^-/-^ (kindly provided by Dr. Patrick Dolph) [44], *Df(Shaker)* (kindly provided by Dr. Kyunghee Koh) [45], *UAS-DNK*_*v*_*4* [40], *UAS-K*_*v*_*4/Shal* [43], *UAS-K*_*v*_*1/Shaker* (kindly provided by Dr. William Joiner), and *UAS-SOD1*, *UAS-SOD2*, *UAS-Catalase*, *UAS-NOX-RNAi* and *UAS-DUOX-RNAi* (all kindly provided by Dr. Matthias Landgraf), *UAS-GFP-K*_*v*_*4/Shal* [46], and *elav-GAL4*, *tub-GAL80*^*ts*^, *UAS-Dcr*^*2*^ (all obtained from the Bloomington *Drosophila* Stock Center).

#### Aging drosophila

Fly stocks were grown at 23–25°C, and male flies < 24 hours AE were collected and housed in vials (10–40 flies per vial, depending on the experiment) at 25°C, 65% humidity for the indicated number of days. Flies were transferred to fresh food every 5–7 days throughout aging periods.

### Digital drop PCR

#### RNA isolation and reverse transcription

Total RNA was extracted from 10 fly heads using TRIzol reagent, treated with DNase I (Thermo Scientific) to remove potential genomic DNA contamination. The integrity of the representative RNA samples was assessed using gel electrophoresis. Total RNA concentration was measured in duplicate using NanoDrop Lite Spectrophotometer (Thermo Scientific) and the purity of the samples was estimated by the OD ratios (A260/A280, ranging within 1.9–2.0). cDNA was synthesized from 700 ng of DNA-free total RNA in a 20 μl reaction volume using SuperScript II RT (Invitrogen) and Oligo (dT) as reverse transcription primers.

#### Primer design and verification

Common sequence from multiple mRNA transcript variants (predicted in Fly Base) were used for PCR primer design. Probe finder version 2.35 and intron spanning assay (Roche) were used to find a proper probe and design primers; Primer3 software was used with the following settings: melting temperatures between 59°C and 61°C, GC content between 40 and 60% and amplicon length limited to 60–200 base pairs. The maximum self-complementarity of the primers was set at 8 and the maximum 3’ complementarity at 3. The PCR primer sets specificity were verified by Primer-Blast (http://www.ncbi.nlm.nih.gov/tools/primer-blast/) using the *Drosophila* transcriptome. Probe 66 was used for *Kv4/Shal* primers (*Left*, GCTAACGAAAGGAGGAACG; *Right*, TGAACTTATTGCTGTCATTTTGC) and *RPS20* primers (*Left*, CGACCAGGGAAATTGCTAAA; *Right*, CGACATGGGGCTTCTCAATA); Probe 147 was used for *eIF1A* primers (*Left*, TCG TCT GGA GGC AAT GTG; *Right*, GCC CTG GTT AAT CCA CAC C). Ribosomal Protein S 20 (RpS20) and Eukaryotic Initiation Factor 1 A (eIF1A) were selected as reference genes based on their stability across experimental conditions. Real-time products were extracted for sequencing and PCR efficiency was calculated from 10-fold serial dilutions of cDNA samples; PCR efficiencies were required to be between 1.9 and 2.0.

#### Droplet generation and PCR

To generate droplets, the 20ul PCR reaction mix and 60ul droplet generation oil were added to wells in a DG8 Cartridge for the QX200 Droplet Generator (Bio-Rad Laboraties). After automated droplet generation, droplets were transferred to a 96-well plate. The plate was sealed with foil using the PX1 PCR Plate Sealer (Bio-Rad Laboratories), and PCR amplification was performed (C1000 Touch Thermal Cycler, Bio-Rad Laboratories). The following thermal cycling protocol was used: 95°C for 10 minutes (one cycle), 94°C for 30 seconds (40 Cycles) and then 60°C for 1 minute (40 cycles), 98°C for 1 minutes (one cycle), hold at 4°C. The ramp rate was set at 2°C/s, the sample volume at 40 mL, and the heated lid at 105°C. After PCR amplification, plates were read in the QX200 Droplet Reader (Bio-Rad Laboratories). Absolute template expression in copies per microliter were quantified using QuantaSoft software (Bio-Rad Laboratories); number of K_v_4/Shal copies/ul were normalized to the number of RpS20 copies/ul from the same RNA sample.

### Immunoblot analysis

#### Drosophila head samples

For each sample, five adult *Drosophila* heads were sonicated in SDS sample buffer (50 mM Tris–HCl, ph 6.8, 10% SDS, glycerol, Dithiothreitol (DTT), bromophenol blue); N refers to the number of samples tested. Proteins were separated on a 10% acrylamide gel. Nitrocellulose blots were probed with primary antibodies overnight at room temperature: anti-Kv4/Shal 1:100; anti-dSK 1:100 was verified with the use of a dSK^-/-^ mutant; anti-K_v_1/Shaker 1:500 (Abcam, Cambridge, MA) was verified with the K_v_1/Shaker *Drosophila* deficiency; anti-actin (Clone C4, MilliporeSigma, MA) 1:2500; anti-syntaxin 1:50 (Developmental Hybridoma Studies Bank). Anti-K_v_4/Shal antibodies were generated as previously described [46, 47]. For mouse brain immunoblots, α-K_v_4.2 (gift from Dr. Michael Tamkun, Colorado State University) at 1:500, α-K_v_4.3 (Neuromab) at 1:500, and α-mActin (Clone AC-40, Sigma-Aldrich, St. Louis, MO) at 1:1000. Blots were incubated with peroxidase-conjugated secondary antibodies (1:2500; Jackson ImmunoResearch Laboratories) for one hour at room temperature, developed using Supersignal Signal^TM^ West Pico PLUS (Thermo Scientific). Anti-K_v_4/Shal, GFP, K_v_4.2 and K_v_4.3 signal densities were normalized to densities from loading control signals (anti-Actin or anti-Syntaxin) from the same lane.

#### Mouse brain samples and immunoblot analysis

Tissue homogenates from noted brain regions from 10 young (6-wk old) and 10 old (8 months old, subsequently aged to 13 months) mice (C57BL/6 from Charles River Laboratories) were obtained from Dr. Robert Handa’s lab (Colorado State University), flash frozen in liquid nitrogen, then stored at -80°C. Prior to each experiment, samples were thawed on ice, protease inhibitors (100X HALT, EDTA free, ThermoFisher Scientific, Waltham, MA) were added to final 1X concentration, and tissue was homogenized using a tissue mincer electric homogenizer. To pellet connective tissue and nuclear material, homogenate was transferred to a 15mL conical vial and spun at 1000x g for 10 minutes at 4°C. Supernatant was then spun at 20,000x g for 15 minutes at 4°C to pellet membrane fraction. Supernatant was removed and pellet re-suspended in 150–300 μL buffer + 1% Triton-X100. Quantification of total protein concentration was performed using the BCA system from Pierce and a UV/Visible spectrophotometer (Model DU730, Beckman Coulter, Brea, CA). Each sample was prepared with 15 μg total protein in 2X SDS-PAGE buffer.

#### Data collection and statistical analysis for mouse brain comparisons

Each experiment was performed at least 5 times. Seven young and seven old (total 14) brain extracts were run on each SDS-PAGE gel. Densitometric analysis, as described above, was performed to quantify anti- K_v_4.2, K_v_4.3 signals, relative to an anti-mActin loading control. A Linear Mixed Effects Model was then used to analyze the effects of age on protein levels of K_v_4 proteins across multiple blots and multiple mice; “age” was used as a fixed effect, “Experimental-Immunoblot” was defined as a variable effect which represents the error across experimental procedures, “Mouse-Brain-Section” was another variable that represents the measurable differences of the same K_v_4 across different mouse brains. Data was fit to this model using the Maximum Likelihood of the “lmer” function in lme4 package [48] of the R statistical software using RStudio (http://www.rstudio.com), setting REML to FALSE (this option is used when comparing different fixed effects which in this case was age). The p-values were calculated from the fixed effects t-values obtained by the “lmer” function on data as a function of age.

Software syntax for the mathematical expression:

$$\text{model}1<-\text{lmer}\left(\text{Data}\sim \text{Age}+\left(1|\text{Experiment}\right)+\left(1|\text{Brain}\right),\text{data}=“\text{datavalues}”,\text{REML}=\text{FALSE}\right)$$

### ROS fluorescence detection

1 mM 2’,7’-dichlorodihydrofluorescein diacetate, H_2_DCFDA (Invitrogen, Walthman, MA) in dimethyl sulfoxide (DMSO; Sigma-Aldrich, St. Louis, MO) was made fresh for each experiment. To calibrate fluorescence gain for each experiment, 1 μL H_2_DCFDA stock was mixed with 1 pM, 1 nM, 1 μM, or 1 mM H_2_O_2_ (Sigma-Aldrich, St. Louis, MO) in separate wells. Samples were prepared by homogenizing 25 heads into 500 μL of sterile filtered buffer (0.4 M Tris-HCL, pH 7.4), centrifuged at 5,000 RPM in a top table centrifuge to pellet chiton. 100 μL samples were loaded into single wells (4 samples per each 500 μL extraction, with at least 5 extractions per experiment) and 1 μL 1 mM DCFDA was added to each well in 96-well plates. Fluorescence Endpoint readings were collected (Synergy H1, BioTek; Excitation 489, Emission: 525); number of extractions, technical replicates, and experimental replicates are indicated in figure legends.

### Drosophila locomotor activity assay

35–40 adult males, aged at 25°C for indicated ages, in a 12.4 cm tall tube were allowed to climb upwards for 30 seconds into a second tube inverted on top of the first. The flies that successfully climbed into the second tube were given 30 seconds to climb from the bottom of the tube into a third tube. This process was continued through ten successive tubes and measured by a countercurrent distribution [49]; each fly was given a score of 0.5 for each tube that it climbed out of, similar to previous studies [40, 43]. 10 assays, with ~35–40 naïve flies, were performed for each genotype tested.

## Results

### K_v_4/Shal and K_v_1/Shaker protein levels are affected by age

**Fig 1:**
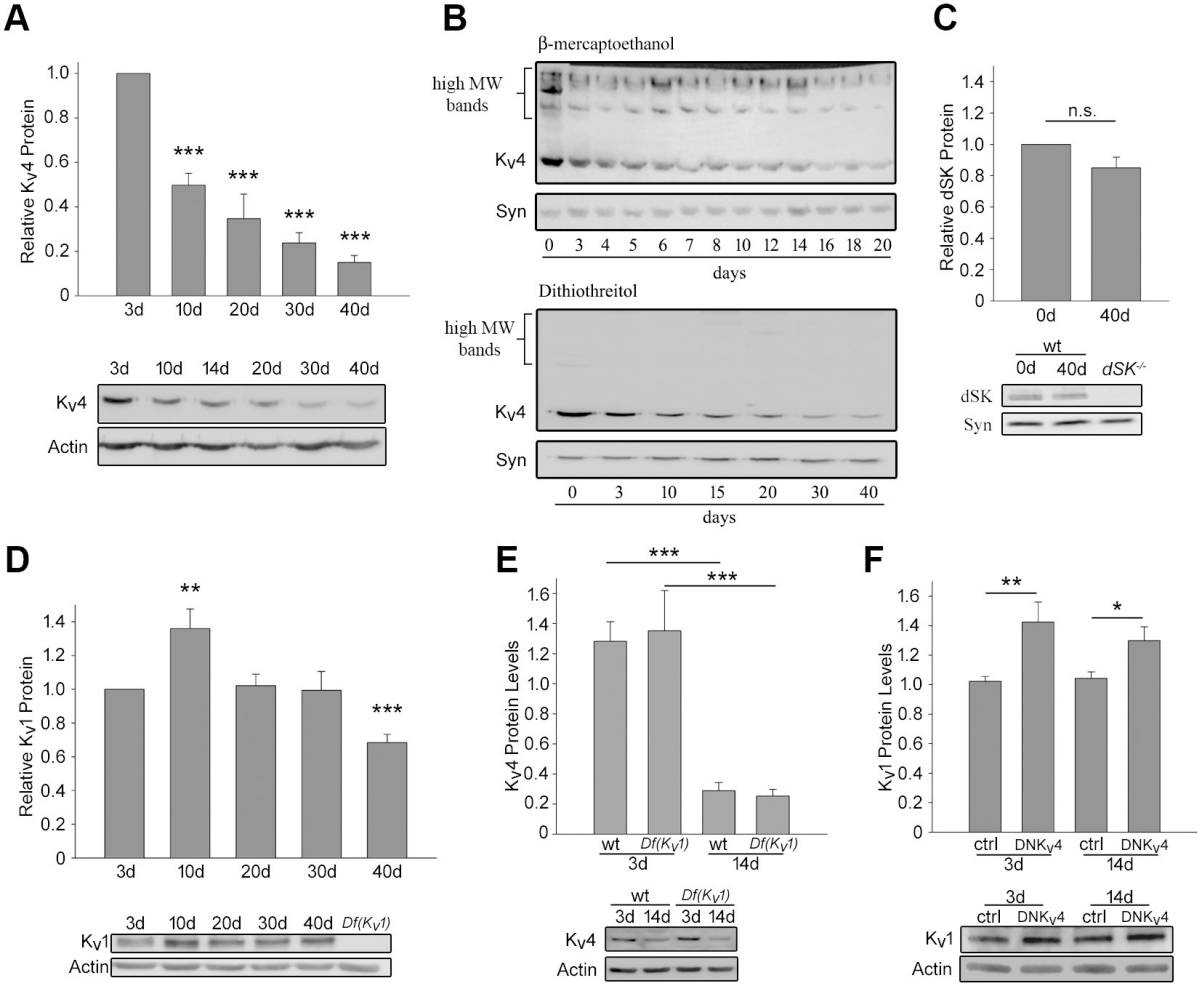
K_v_4/Shal protein levels decline with age, independent of K_v_1/Shaker. Representative immunoblots and quantitative analyses for K_v_4/Shal, dSK, and K_v_1/Shaker, relative to Actin or Syntaxin (Syn) loading controls, as indicated. (A) Representative immunoblots and quantitative analyses (N = 7) of relative K_v_4/Shal levels from wild-type fly heads at indicated days after eclosion. (B) Representative immunoblots of K_v_4/Shal levels from fly head homogenates in sample buffers containing either β-mercaptoethanol (*top*) or the stronger reducing agent, DTT (*bottom*). (C) Relative levels of dSK protein in wild-type heads from newly-eclosed (<24 hours; 0d) and 40 day old flies (N = 10); *dSK*^*-/-*^ null mutant flies were used to verify the anti-dSK antibody. (D) Relative levels of K_v_1/Shaker protein assayed from fly heads of wild-type flies aged as indicated (N = 11); *Df(K*_*v*_*1)* flies were used to verify the anti-K_v_1 antibody. (E) K_v_4/Shal protein levels assayed from wild-type (wt) and *Df(K*_*v*_*1)* fly heads at 3 and 14 days AE, as indicated (N = 12). (F) K_v_1/Shaker protein levels assayed from *elav-GAL4>>UAS-DNK*_*v*_*4* (DNK_v_4) and the *UAS-DNK*_*v*_*4* background control (ctrl) fly heads at 3 and 14 days, as indicated (N = 18). For all immunoblots, 5 fly heads per sample, N indicates the number of samples assayed. Shown are mean values +/- SEM; *p<0.05, ** p≤0.01, *** p≤0.001, Student’s t-test.

To investigate if age affects K_v_4/Shal protein levels, we collected newly-eclosed wild-type male flies (<24 hours old), aged them at 25°C, and assayed steady-state protein levels by immunoblot analysis. We found that K_v_4/Shal protein levels dramatically declined, first by ~50% from 3 days to 10 days after eclosion (AE), then more gradually from 10 days to 40 days to levels less than 20% of those in 3-day old flies (Fig 1A). Since other K^+^ channels have been reported to aggregate with age [27, 50], we investigated if the decline in levels of the expected 52 kD K_v_4/Shal subunit might be accompanied by a rise in levels of a higher molecular weight aggregate. When samples were prepared in an SDS sample buffer containing β-mercaptoethanol as a reducing agent, we did see higher molecular weight bands, but they did not appear to increase with age (Fig 1B), suggesting that there was no correlation with an increase in aggregated K_v_4 protein. For further confirmation, we used a sample buffer containing dithiothreitol (DTT), a stronger reducing agent. We found that the high molecular weight bands were then solubilized, and that the 52 kD K_v_4/Shal band still displayed a progressive age-dependent decline (Fig 1B).

This progressive age-dependent decline in K_v_4/Shal channel protein did not appear to be a consequence for all potassium channels, as protein levels of the *Drosophila* calcium-activated small conductance (SK) potassium channel, dSK, were not significantly different between 0 and 40 days (Fig 1C). Due to the scarcity of antibodies against ion channels in *Drosophila*, we were not able to test other ion channel proteins and cannot rule out the possibility that age may similarly affect other ion channels.

When we examined Kv1/Shaker levels with age, we found that K_v_1/Shaker protein levels exhibited a significant increase from 3 days to 10 days AE that returned to basal levels from 20 to 30 days AE (Fig 1D). The transient increase in K_v_1/Shaker levels between 3 and 10 days of age is concurrent with the steepest decline in K_v_4/Shal levels, consistent with previous studies that have reported an inverse relationship between K_v_1/Shaker and K_v_4/Shal expression [51, 52]. From 30 to 40 days AE, there was, however, a ~30% decline in K_v_1/Shaker protein level, similar to K_v_4/Shal. One possibility is that the early decline in K_v_4/Shal protein from 3 to 10 days AE is a result of the transient increase in K_v_1/Shaker protein from 3 to 10 days AE and its reported reciprocal regulation of *K*_*v*_*4/Shal*. To test if *K*_*v*_*1/Shaker* is required for the observed decline in K_v_4/Shal protein that we observed, we compared relative levels of K_v_4/Shal channel protein in a *Drosophila* line carrying a small deficiency that completely removes the *K*_*v*_*1/Shaker* gene (*Df(K*_*v*_*1))*. We found, however, that total K_v_4/Shal protein levels undergo an age-dependent decline in *Df(K*_*v*_*1)* fly heads similar to wild-type (Fig 1E), suggesting that the progressive decline in K_v_4/Shal protein does not depend on *K*_*v*_*1/Shaker* expression.

Since K_v_1/Shaker levels were, conversely, shown to be inversely regulated by K_v_4/Shal function [51–53], we also tested whether levels of K_v_1/Shaker during this time are dependent on K_v_4/Shal function. We used a transgene encoding a dominant-negative K_v_4/Shal subunit, DNK_v_4, under the control of a *UAS* activation sequence; we have shown that expression of *UAS-DNK*_*v*_*4* driven with the pan-neuronal *elav-GAL4* transgene results in near-abolishment of the K_v_4/Shal current [42, 54]. Indeed, when K_v_4/Shal function was inhibited by expression of DNK_v_4, we found that K_v_1/Shaker expression was up-regulated by ~40% and ~30%, in both 3 day and 14 day old flies, respectively (Fig 1F). Thus, the age-related loss of K_v_4/Shal channels from 3 to 10 days AE may contribute to the transient up-regulation of *K*_*v*_*1/Shaker* expression during this time. The decline in K_v_4/Shal protein from 3 to 14 days AE, however, is independent of *K*_*v*_*1/Shaker* expression.

### Age-related locomotor performance and K_v_4/Shal and K_v_1/Shaker expression

**Fig 2:**
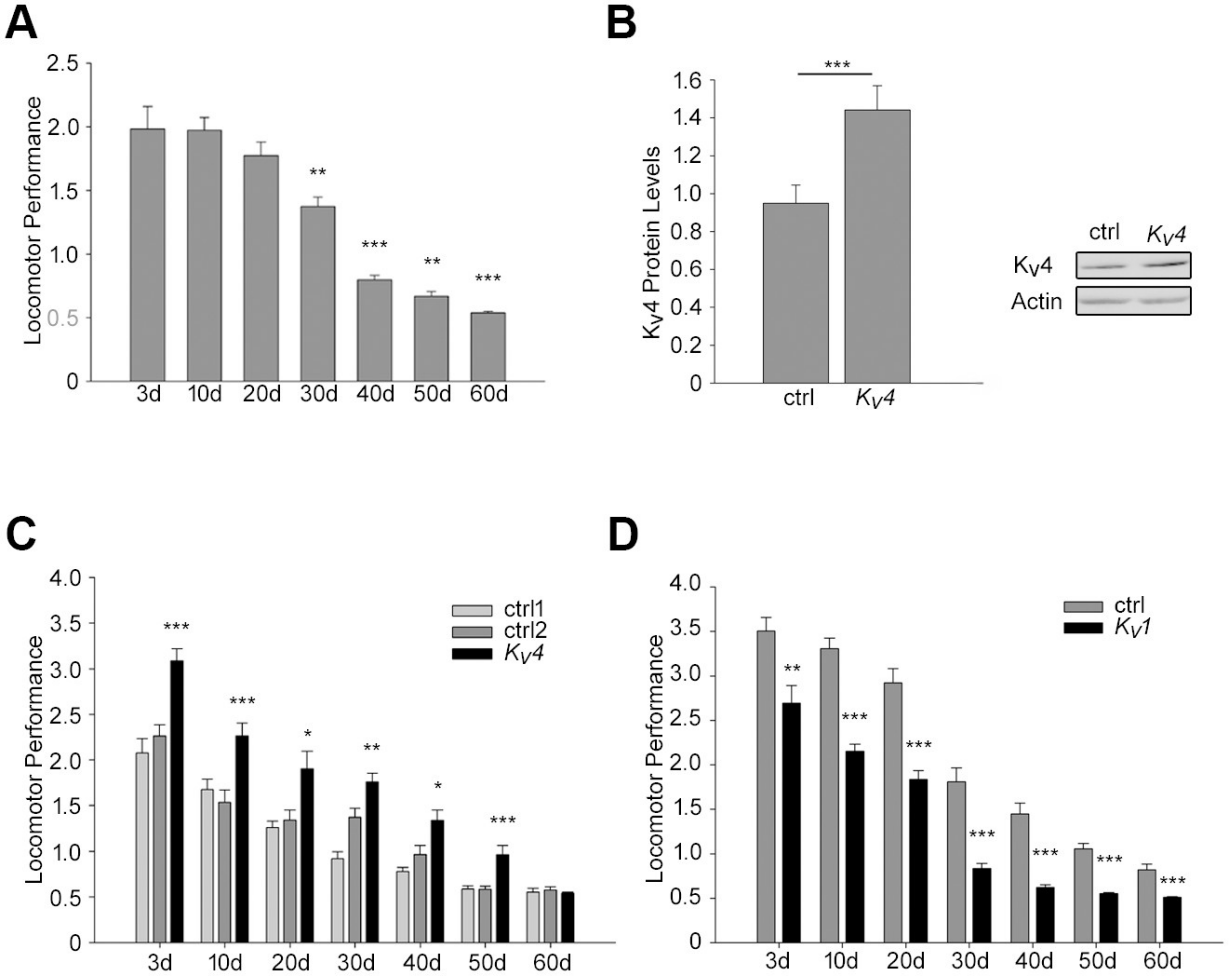
Age-related decline in locomotor performance improved by over-expression of K_v_4/Shal. (A) Locomotor performance scored from climbing assays performed, as described in text, on male wild-type flies aged at 25°C for indicated days. (B) Representative immunoblots and quantitative analyses for K_v_4/Shal protein in heads from *elav-GAL4>>UAS-K*_*v*_*4* (K_v_4) and the *UAS-K*_*v*_*4* background control (ctrl) flies aged 3 days (N = 20). (C) Locomotor performance scored from climbing assays performed on genetic background control lines, *elav-GAL4* (ctrl1) and *UAS-K*_*v*_*4/Shal* (ctrl2), and *elav>>UAS-K*_*v*_*4/Shal* (K_v_4); flies were aged at 25°C for the indicated days. (D) Locomotor performance scored from climbing assays performed on male *UAS-K*_*v*_*1/Shaker* (ctrl) and *elav-GAL4>>UAS-K*_*v*_*1/Shaker* (K_v_1); flies were aged at 25°C for the indicated days. For all assays, 35–40 flies per group, N = 10 groups per genotype were tested for each time point, shown are mean values +/- SEM; *p<0.05, ** p≤0.01, *** p≤0.001, Student’s t-test.

Our previous studies have shown that K_v_4/Shal channels play an important role in repetitive firing and repetitive behaviors [40]. For example, in climbing assays, loss of K_v_4/Shal function results in impaired locomotor performance. Here, we show that K_v_4/Shal channel protein levels in *Drosophila* heads decline with age. One possibility is that this decline represents a general decline in K_v_4/Shal channel protein in all neurons, including neurons in the brain that contribute indirectly to locomotion, and perhaps even neurons outside the brain that control locomotion directly (eg. motor neurons or central pattern generator neurons in the ventral nerve cord). To begin, we examined whether there is an age-dependent decline in locomotor performance in wild-type flies. Wild-type flies aged at 25°C for 3 to 60 days AE were subjected to countercurrent climbing assays in groups of 35–40 male flies per group; they were scored for their ability to climb against gravity through 10 successive tubes (see Materials and methods). Indeed, wild-type flies exhibited a progressive decline in locomotor performance with age (Fig 2A), a phenomenon conserved across species. From 3 to 10 days AE, however, there was no significant decline in motor performance (Fig 2A), suggesting that the sharp initial decline in K_v_4/Shal protein and transient increase in K_v_1/Shaker protein, in the brain, do not affect locomotion. Locomotor performance showed an initial decline at 30 days AE, and this decline continued to 60 days AE (Fig 2A). Since K_v_4/Shal and K_v_1/Shaker protein levels both showed a significant, albeit more gradual, decline after 30 days (Fig 1A), we set out to genetically restore K_v_4/Shal or K_v_1/Shaker levels in aging flies and examine if consequent locomotor performance was improved. We first used the *elav-GAL4* transgene to drive expression of *UAS-K*_*v*_*4/Shal* throughout the nervous system (these flies are referred to as *elav-GAL4>>UAS-K*_*v*_*4/Shal*), and verified that *elav-GAL4>>UAS-K*_*v*_*4/Shal* flies displayed an increase in total K_v_4/Shal protein (Fig 2B). We then compared the locomotor performance of *elav-GAL4>>UAS-K*_*v*_*4/Shal* flies to age-matched genetic background control lines. We found that over-expression of *K*_*v*_*4/Shal* significantly improved locomotor performance not only at 30–50 days AE, but at every age, except at 60 days AE (Fig 2C). These results do not definitively show that the progressive decline in K_v_4/Shal protein underlies the age-related decline in locomotor performance, as there is still an decline over time even when K_v_4/Shal levels are raised. Our results do, however, suggest that the inability to maintain higher levels of K_v_4/Shal with age is a likely contributor. While the decline in K_v_1/Shaker protein from 30 to 40 days AE (Fig 1D) does correlate rather well with the age-related decline in locomotor performance (Fig 2A), over-expression of *K*_*v*_*1/Shaker* did not rescue locomotor performance, but conversely, had a negative impact at every age (Fig 2D). These results suggest that the age-related decline in K_v_1/Shaker observed at 40 days AE is not likely to be a significant contributing factor of the decline in locomotor function.

### Reactive oxygen species negatively affect K_v_4/Shal protein levels, and the age-dependent decline in K_v_4/Shal levels are ameliorated by over-expression of catalase

**Fig 3:**
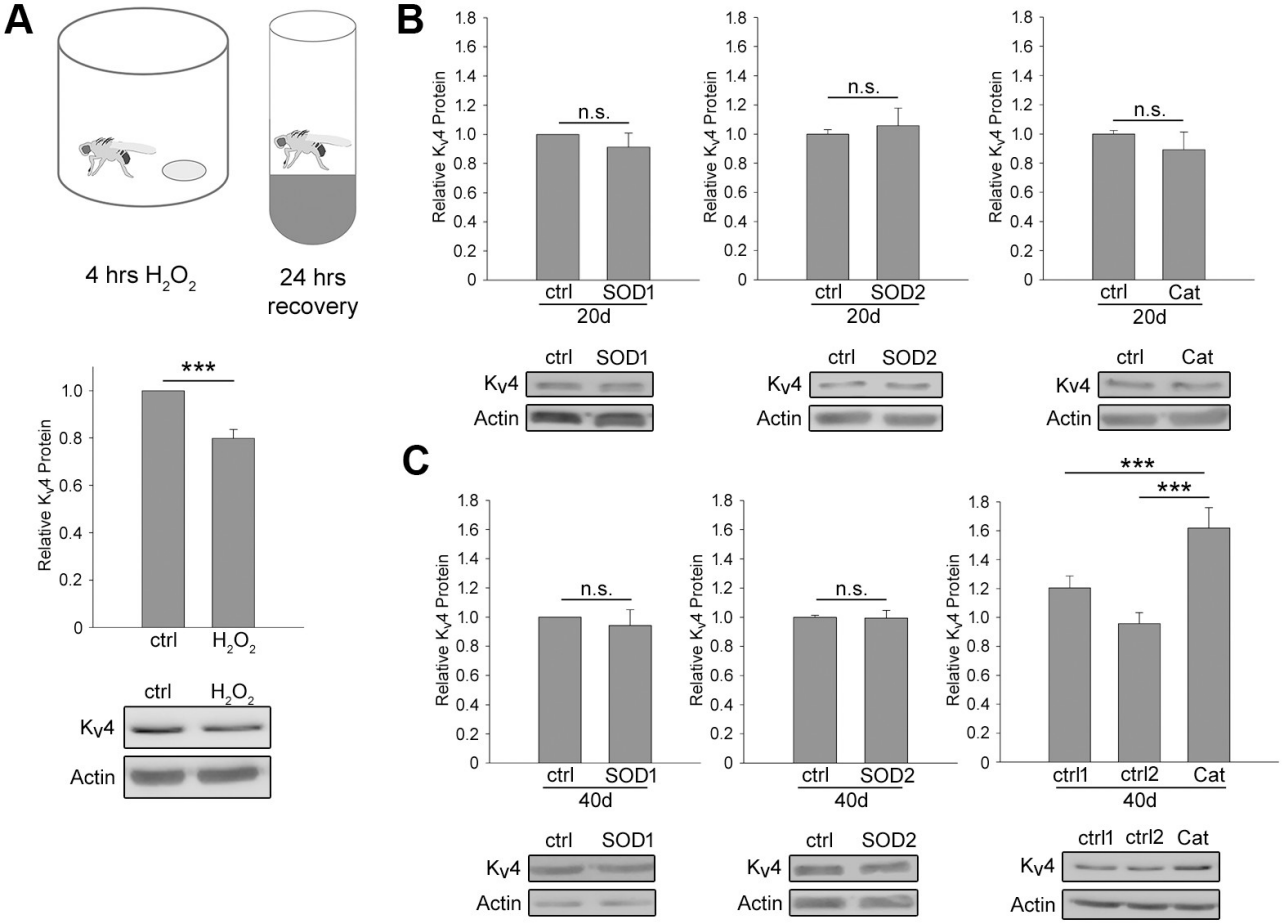
K_v_4/Shal protein levels reduced by ROS and ameliorated by over-expression of catalase in older flies. (A) Acute exposure to hydrogen peroxide (H_2_O_2_) leads to decreased K_v_4/Shal protein levels. *Top*, cartoon depicting incubation of *Drosophila* in a closed scintillation vial with a paper filter containing 100 μL H_2_O_2_, followed by recovery in regular food vial at room temperature. *Bottom*, Representative immunoblots and quantitative analyses of K_v_4/Shal protein levels, normalized to an anti-Actin loading control, in wild-type flies incubated with either water (ctrl) or H_2_O_2_ for 4 hours followed by 24 hours recovery (N = 15). (B-C) Representative immunoblots and quantitative analyses of relative K_v_4/Shal protein levels, normalized to an anti-Actin loading control, in heads from *UAS-SOD1* (*Left*, ctrl), *elav-GAL4>>UAS-SOD1* (*Left*, SOD1), *UAS-SOD2* (*Middle*, ctrl), *elav-GAL4>>UAS-SOD2* (*Middle*, SOD2), *UAS-Catalase* (*Right*, ctrl), *elav-GAL4>>UAS-Catalase* (*Right*, Cat) flies aged at 25°C for either 20 days (B) or 40 days (C). N = 18 samples/genotype and condition. For all immunoblots, 5 heads/sample, N represents the number of samples. Shown are mean values +/- SEM; *p<0.05, ** p≤0.01, *** p≤0.001, Student’s t-test.

K_v_4/Shal protein levels showed a continual decline even after 30 days AE into the very aged fly. One possible underlying factor may be the accumulation of reactive oxygen species (ROS) with age. We first tested whether acute exposure of flies to ROS could affect K_v_4/Shal protein levels. We incubated groups of 30–35 wild-type flies (2–3 days AE) in scintillation vials containing a piece of filter paper saturated with 100 μL of either water or 30% (8.82 M) H_2_O_2_. After 4 hours, flies were transferred to regular food vials for recovery. We found that in flies exposed to H_2_O_2_, K_v_4/Shal levels were lowered by ~20% (Fig 3A), suggesting that K_v_4/Shal protein levels are potentially susceptible to ROS.

To reduce ROS in the aging fly, we over-expressed enzymes well known to reduce ROS *in vivo*. Superoxide dismutase (SOD) 1 and 2, and Catalase actively participate in down-regulating the toxic accumulation of ROS in aging cells. SOD converts superoxide anions to H_2_O_2_, while Catalase converts H_2_O_2_ to H_2_O and O_2_. We tested whether overexpression of SOD1, SOD2, or Catalase would exacerbate or ameliorate the loss K_v_4/Shal protein with age. We used *elav-GAL4* to over-express *UAS-SOD1*, *UAS-SOD2*, or *UAS-Catalase* in neurons, then assayed levels of K_v_4/Shal protein in 20-day old and 40-day old flies. At 20 days AE, no significant change in K_v_4/Shal protein levels were observed with over-expression of any of these enzymes compared to age-matched background control lines (Fig 3B). At 40 days AE, no significant difference in K_v_4/Shal levels was observed with either over-expression of SOD1 or SOD2. Over-expression of *Catalase*, however, significantly increased levels of K_v_4/Shal by ~30 and ~60% when compared to *elav-GAL4* and *UAS-Catalase* background control lines, respectively (Fig 3C). The effect of Catalase, but not SOD1/2, suggests that K_v_4/Shal may be more susceptible to H_2_O_2_ than superoxide anions.

**Fig 4:**
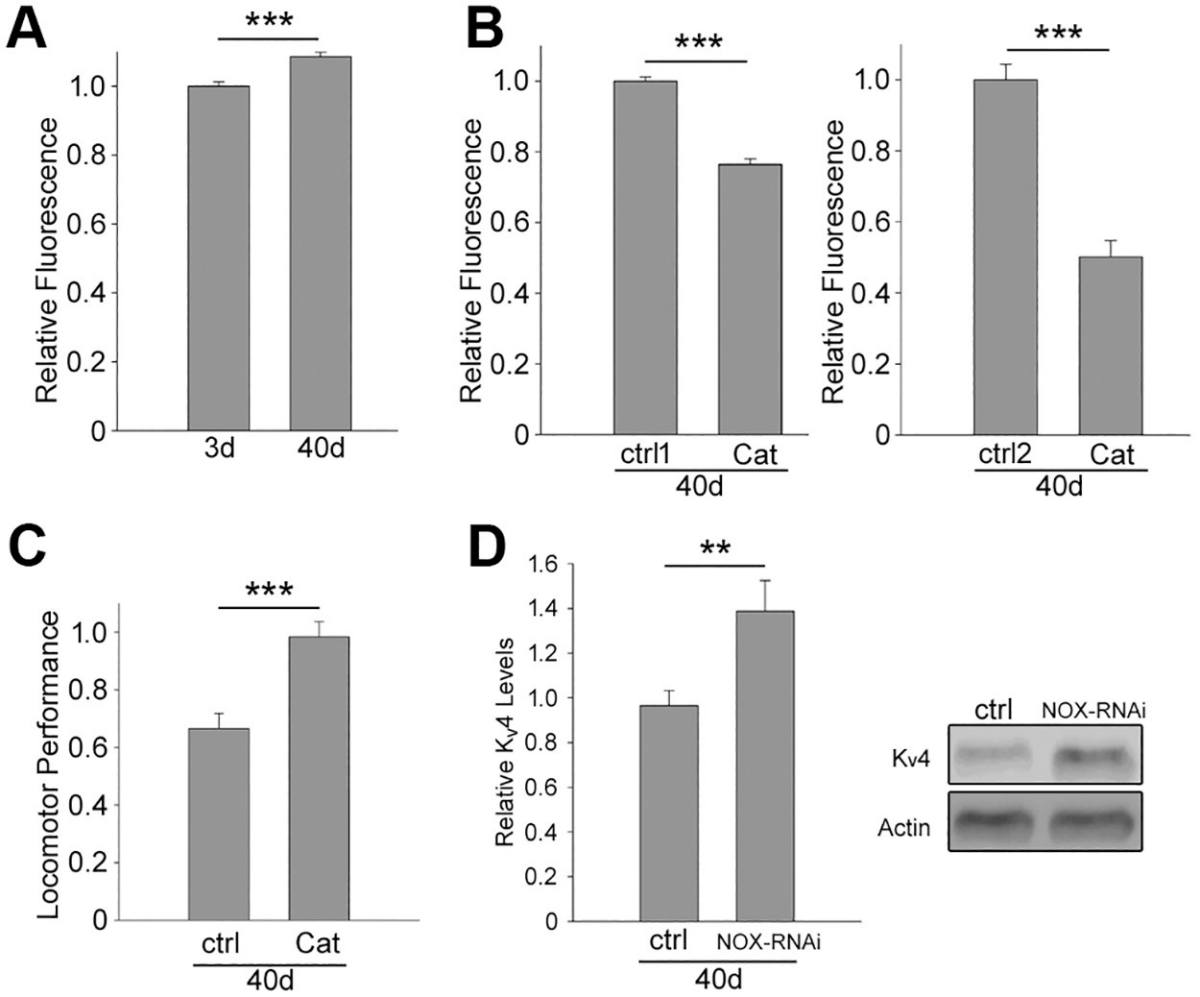
40-day old flies exhibit elevated ROS levels that contribute to impaired locomotion and reduced K_v_4/Shal levels. (A) ROS detected using H_2_DCFDA, a ROS sensitive DCFDA compound, in fly head homogenates by spectrofluorimetry from wild-type flies aged 3 or 40 days at 25°C. 25 heads/extraction, 5 extractions per experiment with 4 technical replicates each; total of 3 experiments were performed. (B) ROS similarly detected, as in (A), in fly head homogenates from *elav-GAL4* (ctrl1), *elav-GAL4>>UAS-Catalase* (Cat), and *UAS-Catalase* (ctrl2) at 40 days AE. 25 heads/extraction, 5 extractions per experiment with 4 technical replicates each; total of 3 experiments were performed. (C) Locomotor performance scored from climbing assays performed on male *UAS-Catalase* (ctrl) and *elav-GAL4>>UAS-Catalase* (Cat) flies at 40 days AE; 35–40 flies/group, N = 10 groups for each genotype. (D) Representative immunoblots and quantitative analysis for K_v_4/Shal relative to anti-Actin as a loading control in heads from 40-day old *UAS-NOX-RNAi* (ctrl) and *elav-GAL4>>UAS-NOX-RNAi* (NOX-RNAi) flies; N = 26. For all immunoblots, 5 heads/sample, N represents the number of samples. Shown are mean values +/- SEM; *p<0.05, ** p≤0.01, *** p≤0.001, Student’s t-test.

Because Catalase expression attenuated the loss of K_v_4/Shal protein at 40 days AE, we tested if *in vivo* levels of ROS are enhanced at 40 days, and if over-expression of Catalase does indeed decrease levels of ROS. We used 2’,7’-dichlorodihydrofluorescein diacetate (H_2_DCFDA), a ROS sensitive DCFDA compound, to detect ROS in fly head homogenates by spectrofluorimetry. Wild-type flies showed a small but significant increase in detectable ROS at 40 days AE compared to 3 days AE (Fig 4A); this small but significant increase in ROS is similar to the previously reported increase ROS at 50 days [6]. We then assayed for ROS levels in *elav-GAL4>>UAS-Catalase* flies at 40 days AE, and found that ROS levels were indeed reduced by ~20% and ~50% when compared to *elav-GAL4* and *UAS-Catalase* background controls, respectively (Fig 4B). Together, our data suggest that at 40 days AE, ROS levels are normally elevated, and that over-expression of *Catalase* results in a reduction in ROS as well as an increase in K_v_4/Shal protein. Since over-expression of *Catalase* raises K_v_4/Shal levels, we tested if over-expression of *Catalase* would also result in improved locomotor performance. We performed climbing assays on 40-day old *elav-GAL4>>UAS-Catalase* and background control lines. We found that, indeed, locomotor performance was improved by ~32% when *Catalase* was over-expressed (Fig 4C). To further investigate the possibility that ROS may contribute to the age-dependent decline in K_v_4/Shal protein and locomotor dysfunction, we tested if the ROS-generating enzyme, Nicotinamide Adenine Dinucleotide Phosphate (NADPH) Oxidase (NOX), might also affect levels of K_v_4/Shal protein. There is only a single NOX gene in the *Drosophila* genome and we used a *UAS-NOX-RNAi* transgene to knockdown expression of NOX. This *UAS-NOX-RNAi* line has been reported to knock-down NOX expression ~60% [55], has been extensively used in the field [56–59], and has been shown to result in blocking a rise in ROS levels when induced in various tissues by various means [56, 59, 60]. We used *elav-GAL4*,*UAS-*Dcr2 to drive expression of *UAS-NOX-RNAi* to knockdown expression of NOX in the nervous system. We then measured levels of K_v_4/Shal protein in 40 day old flies. We found that knockdown of *NOX* resulted in ~40% more K_v_4/Shal protein, compared to age-matched control lines (Fig 4D). Our results suggest that decreasing ROS levels in the aging fly, by either over-expressing *Catalase* or inhibiting expression of *NOX*, attenuates the loss of K_v_4/Shal protein.

### Age-dependent decline in K_v_4/Shal mRNA

**Fig 5:**
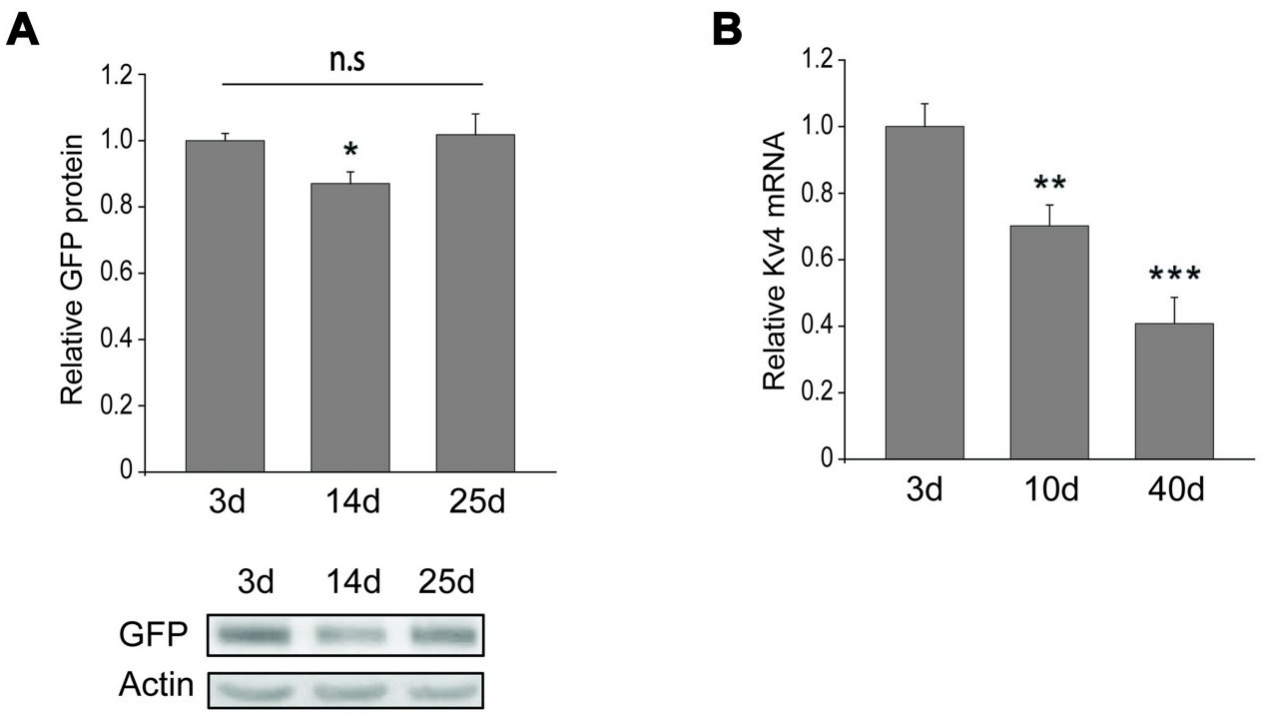
Age-related decline in K_v_4/Shal mRNA, but not GFP-K_v_4/Shal protein. (A) Representative immunoblots and quantitative analysis of GPF-K_v_4/Shal expression from wild-type flies aged 3, 14, and 25 days, normalized to an anti-Actin loading control (N = 43–44). No significant downward trend was observed with age. (B) Digital droplet quantitative PCR was performed for relative *K*_*v*_*4/Shal* levels, normalized to levels of the *RpS20* reference gene, from wild-type flies aged 3, 10, and 40 days. For immunoblot analysis, 5 heads/sample, N represents the number of samples. Shown are mean values +/- SEM; *p<0.05, ** p≤0.01, *** p≤0.001, Student’s t-test.

One possibility is that ROS somehow, directly or indirectly, affect levels of K_v_4/Shal protein. In some cases, ROS have been shown to oxidize protein residues, thereby affecting protein stability, aggregation, and/or degradation. If K_v_4/Shal protein is directly affected by ROS, we reasoned that K_v_4/Shal protein expressed from a transgene would also be affected by age. We used *elav-GAL4* to drive expression of *UAS-GFP-K*_*v*_*4/Shal* pan-neuronally, collected newly-eclosed flies and aged them at 25°C. We then compared steady-state GFP-K_v_4/Shal protein levels at 3, 14, and 25 days AE. In contrast to endogenous K_v_4/Shal levels, we found no significant decline in GFP-K_v_4/Shal protein levels from 3 to 25 days (Fig 5A), suggesting that the age-dependent decline in K_v_4/Shal protein does not occur as a result of the protein being targeted for degradation.

ROS have also been reported to affect gene expression by damaging nucleic acids. To examine if mRNA levels of *K*_*v*_*4/Shal* were affected with age, we performed Digital Droplet PCR to quantify mRNA levels in wild-type heads at 3, 10, and 40 days AE. We found that the number of copies of *K*_*v*_*4/Shal* mRNA was progressively reduced, by ~30% at 10 days and ~60% at 40 days, relative to levels at 3 days AE (Fig 5B). These results suggest that the age-dependent decline in total K_v_4/Shal protein is at least partially due to a progressive decline in *K*_*v*_*4/Shal* mRNA. Future studies will need to examine whether there is any specificity to this effect.

### Mouse K_v_4.2 and K_v_4.3 are differentially affected by age in select brain regions

**Fig 6:**
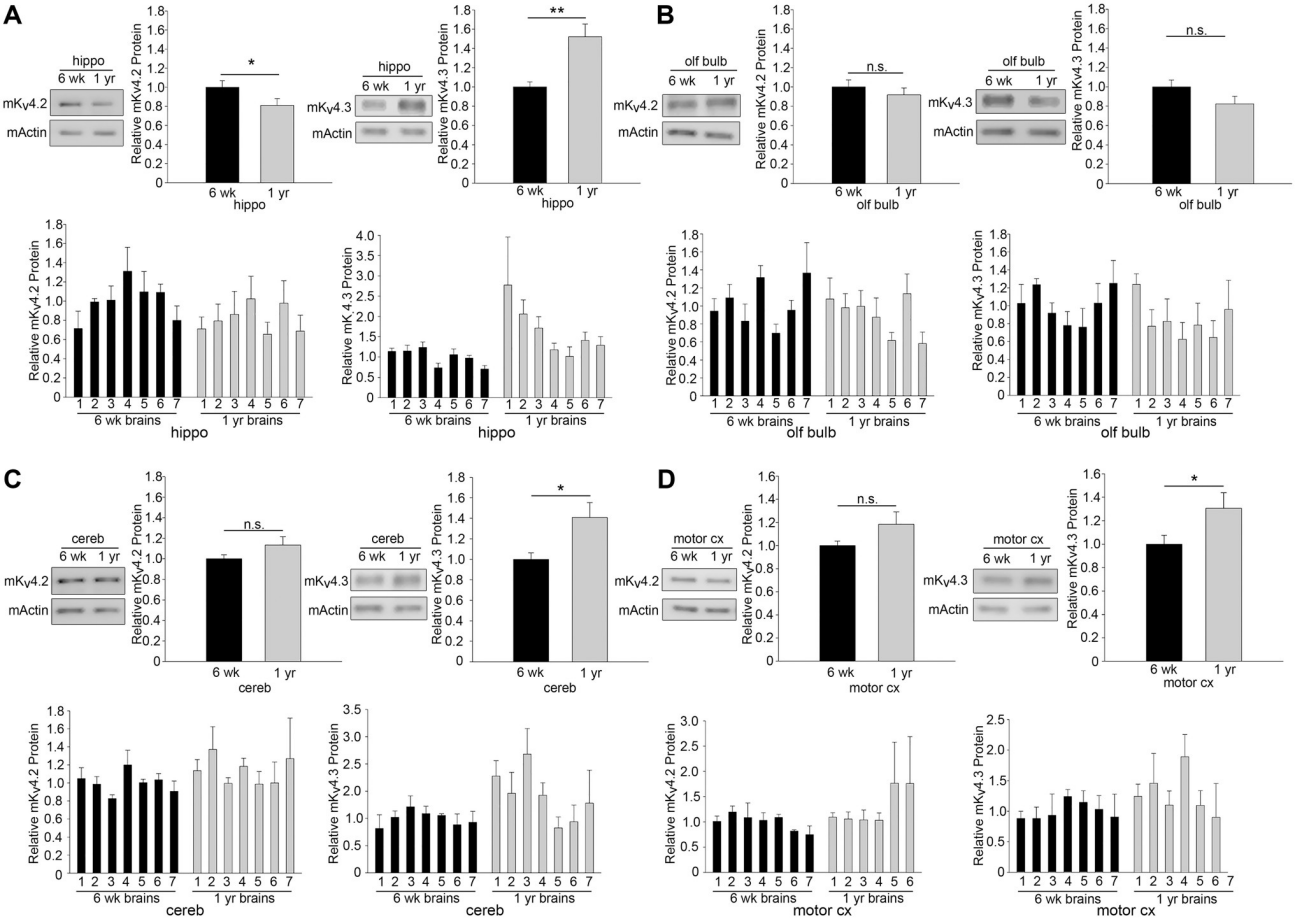
Differential effects of age on mouse K_v_4.2 and K_v_4.3 in various brain regions. Representative immunoblots and quantitative analysis, as described in the text, for K_v_4.2 and K_v_4.3 protein in tissue homogenates from seven 6-wk old mice and seven 13-month old mice; tissue homogenates are from the (A) hippocampus (hippo), (B) olfactory bulb (olf bulb), (C) cerebellum (cereb), and (D) motor cortex (motor cx). Black bars represent 6-week (6wk) old mice and grey bars represent 13-month (1yr) old mice. 15 μg total protein per sample was loaded into each SDS-PAGE well (N = 35 samples, across 5 blots, for each brain at each age). *Top graphs*, shown are mean values +/- SEM across 7 brains; *Bottom graphs*, shown are mean values +/- SEM from individual brains; *p<0.05, ** p≤0.01, Linear Mixed Model statistical analysis (see Materials and methods).

K_v_4/Shal channels are highly conserved, with 84% amino acid identity between *Drosophila* and mouse/human (eg. sequence alignment with mouse K_v_4.1 (NP_032449.1), K_v_4.2 (NP_062671.1), K_v_4.3 (NP_001034436), or human K_v_4.2 (NP_036413.1); [30]). As such, we set out to examine if age also affects levels of K_v_4 channel proteins in the mouse brain. We measured steady-state levels of K_v_4.2 and K_v_4.3 in different brain regions, including the hippocampus, olfactory bulb, cerebellum, and motor cortex, where they have been shown to play important roles in neuronal signaling [61–65]. We examined these brain regions in 14 mice: seven at 6 weeks of age and seven at 13 months of age. Anti-K_v_4.2 and K_v_4.3 signals relative to a loading control protein were normalized to the young samples, then compared across multiple immunoblots using Linear Mixed Model (LMM) statistical analysis to account for multiple mice at two different ages, across multiple blots. We found that K_v_4.2 and K_v_4.3 were differentially affected by age in various brain regions. For example, from 6 weeks to 13 months, K_v_4.3 exhibited a ~30–50% increase in the hippocampus, cerebellum, and motor cortex, while K_v_4.2 exhibited a ~20% reduction in the hippocampus (Fig 6). While there was no unified down-regulation of K_v_4.2/4.3 with age that paralleled what we report in *Drosophila*, such a survey of changes in K_v_4.2 and K_v_4.3 in the mouse brain, to our knowledge, has not been reported previously and may be informative for future studies examining their contribution to changes in cognitive and motor function that occur with age.

## Discussion

Physiological effects associated with aging and age-related diseases, such as a decline in motor and cognitive function, are undoubtedly due to a multitude of factors. Here, we report that in *Drosophila*, K_v_4/Shal levels sharply decline from 3 to 10 days AE, then continue a more gradual and progressive decline from 10 to 40 days AE. In contrast, K_v_1/Shaker protein exhibits a transient increase at 10 days AE, that then remains relatively stable until an eventual decline at 40 days AE. It should be noted that 40 days AE is still considered middle-aged in a lifespan of laboratory raised flies that can reach 80 days [66]. As such, these changes in K_v_4/Shal and K_v_1/Shaker levels in the young to middle-aged fly are likely to have physiologically relevant functional consequences. For example, K_v_4/Shal studies in *Drosophila* have shown that loss of K_v_4/Shal channels leads to increased neuronal excitability with faster response times to action potential (AP) firing, increased AP firing rates, enhanced synaptic currents, and altered synaptic plasticity mechanisms [41, 42, 54]. Behaviorally, loss of K_v_4/Shal function leads to loss of normal coordination in repetitive behaviors, such as locomotion and grooming, significantly reduced performance in olfactory-associative learning tasks, and a shortened lifespan [54].

In this study, we show an age-dependent deterioration in locomotor performance. Interestingly, genetic over-expression of *K*_*v*_*4/Shal*, but not *K*_*v*_*1/Shaker*, improved locomotor performance. This relationship, however, is complicated and much remains to be understood. For example, the sharpest decline in K_v_4/Shal protein occurs from 3 to 10 days AE, yet there was no deterioration in locomotor performance until 30 days AE. It is possible that locomotor performance is quite robust and is only affected after K_v_4/Shal protein levels decline below some threshold. At 40 days AE, K_v_1/Shaker levels are also significantly reduced, and perhaps the combinatorial loss of both channel proteins adversely affects performance. Also interesting is the finding that even though over-expression of K_v_1/Shaker appeared to negatively affect locomotion, the transient increase in K_v_1/Shaker at 10 days does not seem to change locomotor performance. Future studies will need to address why locomotor performance at younger ages (3–10 days) remains stable in the face of changing K_v_1/Shaker and K_v_4/Shal levels. It is also possible that changes in K_v_1 and K_v_4 protein levels are entirely different in the ventral nerve cord, where motor neurons and central pattern generator neurons are localized and contribute most directly to locomotion, and future studies may reveal differential, neuron-specific, effects of age on channel expression levels.

In a previous study, we reported that K_v_4/Shal channel levels also progressive decline in a *Drosophila* model of Alzheimer’s Disease (AD) [43]. Mammalian AD models have similarly suggested a decrement in K_v_4 currents/channels [67, 68]. Loss of K_v_4/Shal protein in the *Drosophila* AD model led to neuronal hyperexcitability that contributed to a decline in cognitive and motor function, as well as neuronal degeneration, when compared to age-matched background lines [43]. Early increases in neuronal excitability have been observed in other AD models as well [69–76], and early hyperactivity has been suggested to contribute to downstream synaptic silencing [77] and neurodegeneration [78, 79]. It is intriguing that K_v_4/Shal channel levels are also affected by age in wild-type flies, as reported here. One speculation is that age-affected proteins, such as K_v_4/Shal, are especially susceptible in backgrounds prone to AD, and perhaps other age-related neurodegenerative diseases.

Understanding the mechanism(s) underlying the age-dependent loss of K_v_4/Shal protein may give important insight into not only how K_v_4/Shal protein is targeted under normal aging conditions, but also how it becomes a target in AD and perhaps other neuropathological conditions. We show that protein levels of GFP-K_v_4/Shal expressed from a transgene are stable and not affected by age. Additionally, we show that there is an age-dependent decline in *K*_*v*_*4/Shal* mRNA levels. These data suggest that relevant age-dependent pathway(s) may be affecting *K*_*v*_*4/Shal* expression prior to protein translation. Future studies will need to investigate this possibility.

We also suggest a possible role for ROS, which are well known to accumulate both with age and AD. ROS have been shown to have a multitude of effects on proteins as well as nucleic acids [25, 80–82]. At 40 days AE, we found that ROS levels were elevated by a small but significant amount in fly heads, similar to a previous report [6]. At this age, we also found that over-expression of *Catalase* or down-regulation of NOX was sufficient to raise protein levels of K_v_4/Shal. Surprisingly, over-expression of *SOD1/2* did not. One possibility is that there was not high enough expression of SOD1/2. Another possibility is that H_2_O_2_ in particular, is a key contributor to the reduction in K_v_4/Shal expression and since Catalase is known to catalyze H_2_O_2_ into water and oxygen, over-expression of *Catalase* was the only enzyme that attenuated the loss of K_v_4/Shal protein. Consistent with the hypothesis that *K*_*v*_*4/Shal* is sensitive to H_2_O_2_, we also show that acute exposure of young flies to H_2_O_2_ was sufficient to reduce K_v_4/Shal protein levels. It is not clear, however, that this decline occurs by the same pathway as during aging. Another complication is that K_v_4/Shal levels decline the most dramatically from 3 to 10 days AE, when ROS levels are not significantly elevated. One possibility is that ROS are a contributing mechanism only to the later more gradual decline in K_v_4/Shal observed, while the earlier decline in K_v_4/Shal is mediated by a different mechanism. Another, not mutually exclusive, possibility is that local microdomains of elevated ROS levels may function in a more targeted mechanism of regulation; more sophisticated approaches are needed to investigate this possibility. It also remains unclear whether the effect of ROS is specific to K_v_4/Shal channel expression or if ROS also affects the expression of other ion channels during aging. Future studies should also investigate whether ROS-mediated mechanisms are physiologically employed to regulate K_v_4/Shal channel expression in young neurons/flies.

In this study, we also take a first-pass survey at whether K_v_4.2 and K_v_4.3 protein levels are affected in different regions of the aging murine brain. From 6 weeks to 13 months, we found that K_v_4.2 and K_v_4.3 levels exhibit age-dependent up- and down-regulation in various regions of the brain. Interestingly, K_v_4.2 only exhibited an age-related decline in the hippocampus. In contrast, K_v_4.3 exhibited only age-dependent increases that were observed in multiple brain regions, including the hippocampus, cerebellum, and motor cortex. Finer dissection of these brain regions will reveal more precisely where these differential regulatory events occur; for example, a previous report has suggested that K_v_4.2 and K_v_4.3 are up-regulated in CA3 pyramidal neurons of the hippocampus [22].

In this study, we examined K_v_4/Shal protein levels from whole *Drosophila* heads, in which large neuronal populations include the Kenyon cells of the mushroom bodies, neurons of the optic lobe, and photoreceptor cells. It is unclear whether the decline in K_v_4/Shal protein we observed occurs similarly across all of these populations, or preferentially in particular neuronal populations. Future studies may reveal age-dependent changes in K_v_4/Shal and K_v_1/Shaker expression that are specific to particular brain structures, and results from such studies would greatly aid in identifying the functional consequences of age-related changes.

## Supporting information

S1 Raw images Uncropped blot images for the indicated figures. (DOCX)
