# Supplementary material for: Age-related changes in Kv4/Shal and Kv1/Shaker expression in Drosophila and a role for reactive oxygen species
Source: PLoS One. 2021 Dec 21;16(12):e0261087. doi: 10.1371/journal.pone.0261087 (PMC8691634; doi:10.1371/journal.pone.0261087)

**S1 Raw Images.** Uncropped blot images for the indicated figures.

Figure 1A: Original blots for anti-Kv4 (left, ~50 kD) and anti-actin (right, ~42 kD).







Figure 1B (Top): Original blots for anti-Kv4 (left, ~50 kD) and anti-syntaxin (right, ~34 kD).







Figure 1B (Bottom): Original blots for anti-Kv4 (left, ~50 kD) and anti-syntaxin (right, ~34 kD).








Figure 1C: Original blots for anti-dSK (left, ~125 kD) and anti-syntaxin (right, ~34 kD).





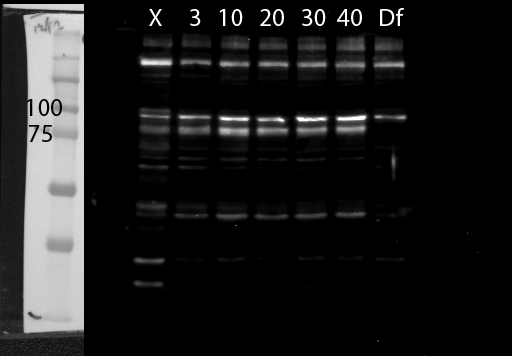
Figure 1D: Original blots for anti-Kv1 (left) and anti-actin (right, ~42 kD).


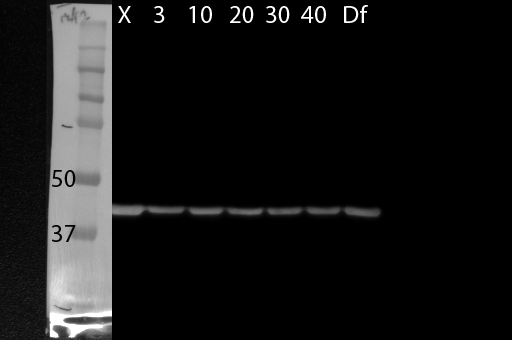


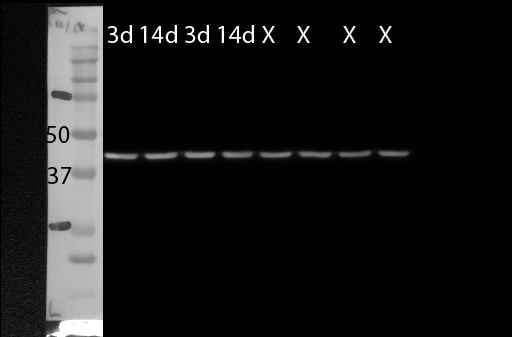

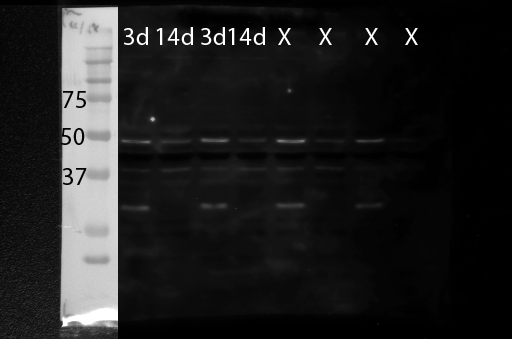
Figure 1E: Original blots for anti-Kv4 (left, ~50 kD) and anti-actin (right, ~42 kD).


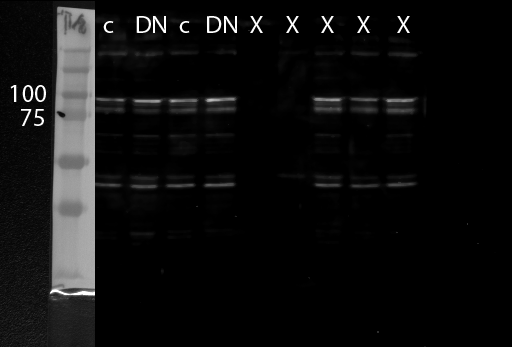
Figure 1F: Original blots for anti-Kv1 (left) and anti-actin (right, ~42 kD).


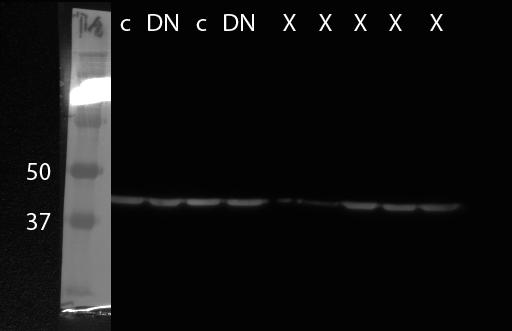


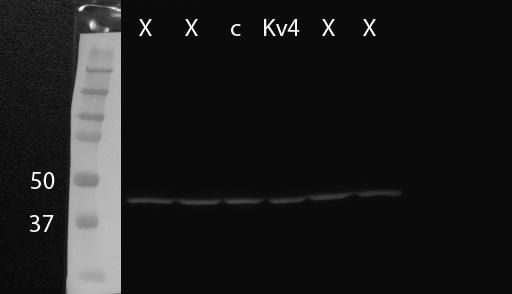

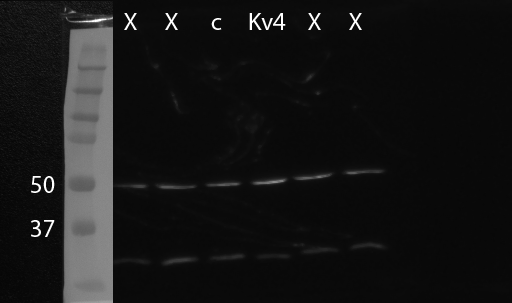
Figure 2B: Original blots for anti-Kv4 (left, ~50 kD) and anti-actin (right, ~42 kD).



Figure 3A: Original blots for anti-Kv4 (left, ~50 kD) and anti-actin (right, ~42 kD).






Figure 3B (Left): Original blots for anti-Kv4 (left, ~50 kD) and anti-actin (right, ~42 kD).


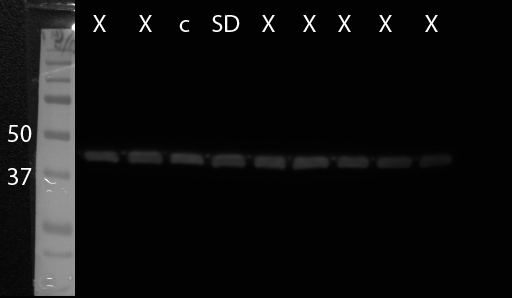



Figure 3B (Middle): Original blots for anti-Kv4 (left, ~50 kD) and anti-actin (right, ~42 kD).






Figure 3B (Right): Original blots for anti-Kv4 (left, ~50 kD) and anti-actin (right, ~42 kD).





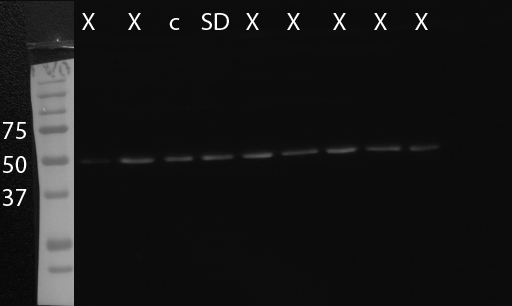
Figure 3C (Left): Original blots for anti-Kv4 (left, ~50 kD) and anti-actin (right, ~42 kD).






Figure 3C (Middle): Original blots for anti-Kv4 (left, ~50 kD) and anti-actin (right, ~42 kD).






Figure 3C (Right): Original blots for anti-Kv4 (left, ~50 kD) and anti-actin (right, ~42 kD).






Figure 4D: Original blots for anti-Kv4 (left, ~50 kD) and anti-actin (right, ~42 kD).





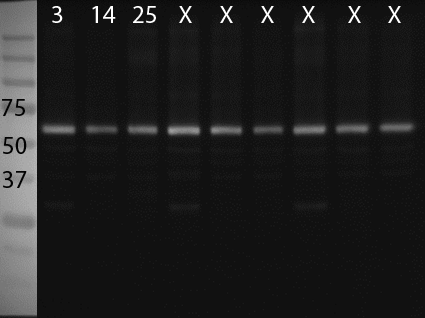
Figure 5A: Original blots for anti-GFP-Kv4 (left) and anti-actin (right).


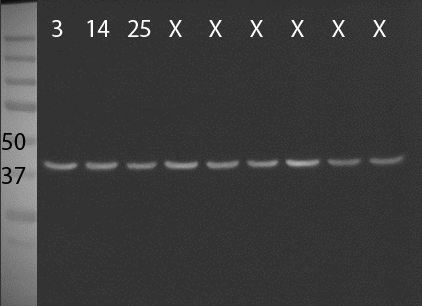





Figure 6A (Left): Original blots for anti-Kv4.2 (left) and anti-actin (right).



Figure 6A (Right): Original blots for anti-Kv4.3 (left) and anti-actin (right).





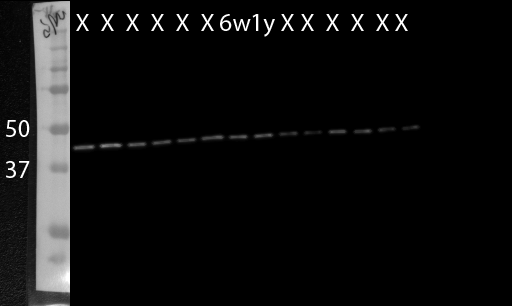


Figure 6B (Left): Original blots for anti-Kv4.2 (left) and anti-actin (right).





Figure 6B (Right): Original blots for anti-Kv4.3 (left) and anti-actin (right).



Figure 6C (Left): Original blots for anti-Kv4.2 (left) and anti-actin (right).






Figure 6C (Right): Original blots for anti-Kv4.3 (left) and anti-actin (right).






Figure 6D (Left): Original blots for anti-Kv4.2 (left) and anti-actin (right).





Figure 6D (Right): Original blots for anti-Kv4.3 (left) and anti-actin (right).




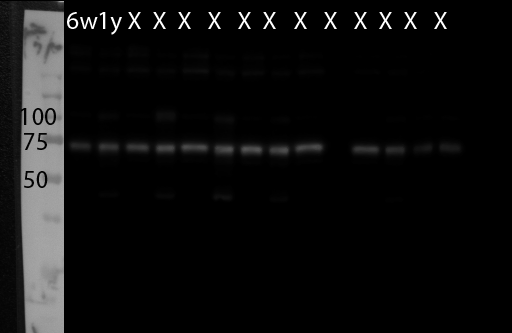

Supplement: S1 Raw images — (DOCX) [file pone.0261087.s001.docx]
